# Assessing Molecular Epidemiology of Carbapenem-resistant *Klebsiella pneumoniae* (CR-KP) with MLST and MALDI-TOF in Central China

**DOI:** 10.1038/s41598-018-38295-8

**Published:** 2019-02-19

**Authors:** Xiujuan Meng, Jun Yang, Juping Duan, Sidi Liu, Xun Huang, Ximao Wen, Xin Huang, Chenchao Fu, Jie Li, Qingya Dou, Yao Liu, Jia Wang, Qun Yan, Mingxiang Zou, Wenen Liu, Zhong Peng, Liang Chen, Chunhui Li, Anhua Wu

**Affiliations:** 1 Infection Control Center, Xiangya Hospital of Central South University, Changsha, Hunan Province China; 2 Bioyong Technologies Inc, Beijing, China; 3 Department of Clinical Laboratory, Xiangya Hospital, Central South University, Changsha, Hunan 410008 China; 4 State Key Laboratory of Agricultural Microbiology, College of Veterinary Medicine, Huazhong Agricultural University, Wuhan, Hubei China; 5 Public Health Research Institute Tuberculosis Center, New Jersey Medical School, Rutgers University, Newark, New Jersey USA

## Abstract

Carbapenem-resistant *K*. *pneumoniae* (CR-KP) posts significant public health challenge worldwide. The aim of this study is to assess clinical characteristics and molecular epidemiology of CR-KP infections with Multilocus sequence typing (MLST) and Matrix-assisted laser desorption ionization–time of flight mass spectrometry (MALDI-TOF) in Central China. A total of 71 CR-KP isolates were recovered in a teaching hospital from October 2014 to December 2015. Among all CR-KP isolates, 73.2% (52) produced *K*. *pneumoniae* carbapenemases-2 (KPC-2). Eighteen ST types were identified by MLST, among these ST types, forty-seven isolates belonged to ST11 type, which was the predominant outbreak strain in China, and most ST11 isolates produced KPC-2. Eleven mass spectrometry (MS) types were identified by MALDI-TOF MS analysis, 53.5% isolates were MS4 and MS6, which matched with ST11 in MLST analysis. CR-KP infection was associated with increased medical cost and longer hospitalization. Therefore, we found that KPC-2-producing ST11 (MS4 and MS6) CR-KP isolates were the predominant clone identified by MLST and MALDI-TOF, and CR-KP infection was associated with increased hospital costs and longer hospitalization.

## Introduction

*K*. *pneumoniae* causes healthcare-associated infections (HAIs), especially in newborns, hematological malignancies patients, and immunocompromised patients^1,2^. Carbapenems are often used to treat Extended-spectrum β-lactamases (ESBL) *K*. *pneumoniae* infection^3^. However, the prevalence of carbapenem-resistant *K*. *pneumoniae* (CR-KP) has risen in recent years, and CR-KP has become a significant public health challenge worldwide^4–6^.

The resistance of *K*. *pneumoniae* to carbapenems is rendered by several mechanisms, including the production of carbapenemases. *K*. *pneumoniae* carbapenemases (KPCs) were originally identified in the USA in 1996^7,8^. Since 1996, carbapenemase genes have spread internationally among *Enterobacteriaceae*, especially *K*. *pneumoniae*. In China, the majority of CR-KP strains acquire resistance to carbapenem by producing KPCs^9–11^. KPC-producing organisms are clinically important because of the limited treatment options available and the high mortality rate caused by these organisms infection.

Interestingly, the geographic distribution of CR-KP in 2013 revealed high incidence of CR-KP around the Yangtze River, covering East and Central regions of China^12^. Zheng B *et al*. studied the molecular epidemiology of CR-KP in Eastern China using Pulsed Field Gel Electrophoresis (PFGE)^13^, however, data on the epidemiology and molecular characteristics of CR-KP infection in central China are lacking, especially, molecular epidemiology of CR-KP using matrix-assisted laser desorption ionization–time of flight mass spectrometry (MALDI-TOF MS). Bacterial identification based on spectra obtained by MALDI-TOF MS developed in the late 1980s, and MALDI-TOF MS is first used to type yeast strains in 2001, has pvoed to be an economical, rapid, and accurate method for typing pathogens^14,15^.

The aim of the present study was to investigate the molecular epidemiology and clinical characteristics of 71 CR-KP isolates in a teaching hospital in Changsha, central China using MALDI-TOF MS and MLST. In addition, this study identified antimicrobial resistance genes of CR-KP strains, and investigated the financial burden of CR-KP infection.

## Results

### Isolates description

**Figure 1:**
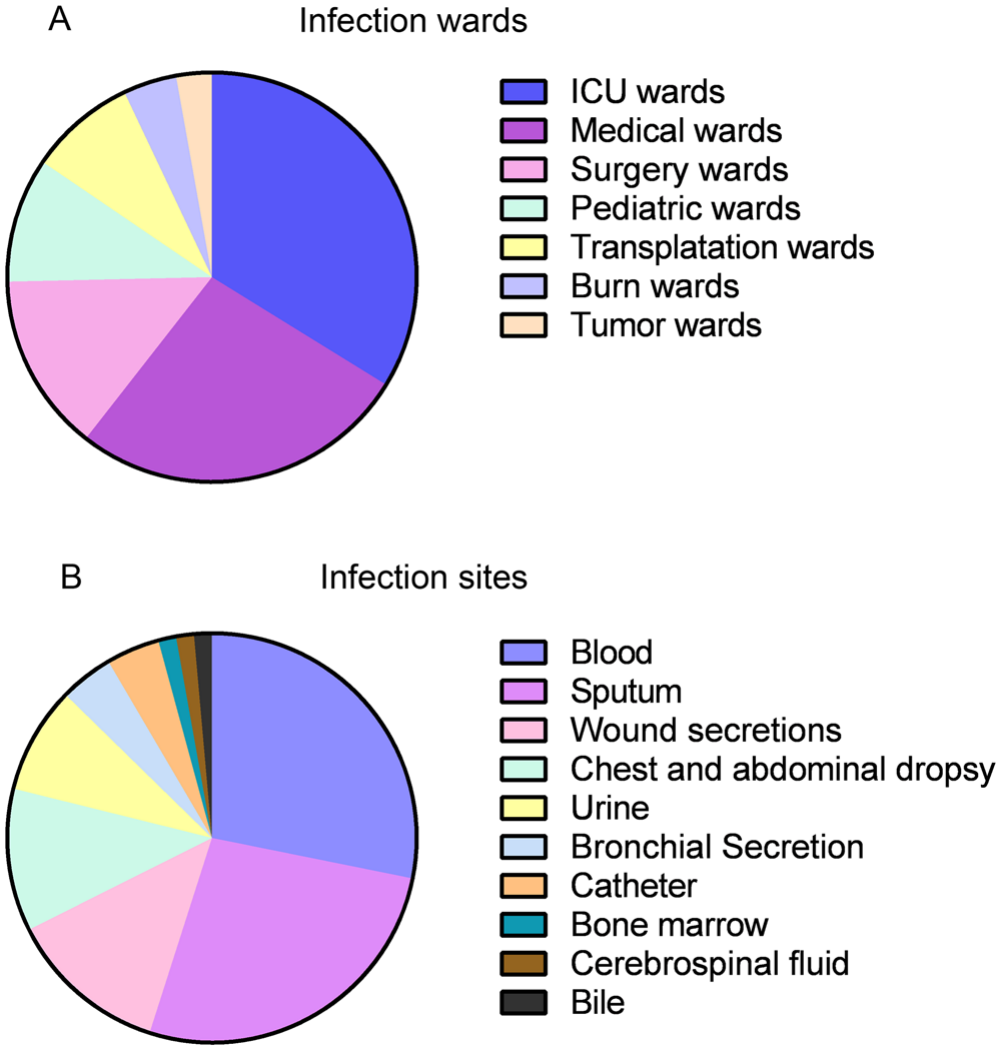
The distribution of carbapenem-resistant *K*. *pneumoniae*. The percentage of carbapenem-resistant *K*. *pneumoniae (*CR-KP) strains recovered from different wards and different sites are presented in this figure. Categorical variables in the figure are (no., %), ICU = intensive care unit.

A total of 71 CR-KP isolates were recovered from hospitalized patients. Among these patients, 46 (64.8%) patients were male and 25 (35.2%) patients were female. The majority of patients with CR-KP infection were from the intensive care unit (ICU) wards (24) and medical wards (19), followed by surgery wards (10), pediatric wards (including neonatal ICU) (7), transplantation wards (6), burn wards (3) and tumor wards (2). The majority of the isolates were recovered from blood (20) and sputum (19), followed by wound secretion (9), chest and abdominal dropsy (8), bronchial secretion (3), catheter (3), bone marrow (1), cerebrospinal fluid (1) and bile (1) (Fig. 1).

### Antimicrobial Susceptibility Test

The antimicrobial susceptibility test results of the CR-KP and CS-KP isolates are shown in Table 1. Approximately 95% of CR-KP strains were resistant to cefoxitin, amoxillin/clavulanic acid, piperacillin/tazobactam, ampicillin/sulbactam, cefazolin, ceftazidime, ceftriaxone, and nitrofurantoin, followed by cefepime, aztreonam, cefotetan, cefoperazone/sulbactam, tobramycin, gentamycin, ciprofloxacin, levofloxacin, and amikacin. Overall, all CR-KP isolates remained susceptible to trimethoprim-sulfamethoxazole.

**Table 1:** The antibiotic-resistance of the two groups {carbapenem-resistant KP (CR-KP) and carbapenem-susceptible KP}.

	CR-KP(n = 71)	CS-KP(n = 71)	OR(95%CI)	*p*
ESBL	5/71 (7%)	33/71 (46%)	28.17 (0.03–0.24)	>0.001
Piperacillin/sazobactam	66/69 (96%)	4/71 (6%)	113.42 (79.39–1710.42)	>0.001
Ampicillin/sulbactam	63/64 (98%)	29/64 (45%)	44.68 (9.93–582.33)	>0.001
Cefoperazone/sulbactam	63/69 (91%)	3/71 (4%)	106.49 (57.09–992.20)	>0.001
Amoxillin/clavulanic acid	6/6 (100%)	5/7 (71%)	2.03 (0.88–2.24)	0.16
Cefazolin	67/69 (97%)	40/70 (57%)	31.31 (5.69–110.81)	>0.001
Ceftazidime	62/64 (97%)	16/65 (25%)	70.44 (20.83–432.76)	>0.001
Ceftriaxone	69/71 (97%)	35/71 (49%)	41.54 (8.07–156.02)	>0.001
Cefoxitin	6/6 (100%)	3/7 (43%)	4.95 (0.99–5.49)	0.03
Cefepime	67/71 (94%)	14/71 (20%)	80.73 (21.25–218.84)	>0.001
Cefotetan	60/65 (92%)	1/67 (1.5%)	109.47 (89.95–6973.50)	>0.001
Aztreonam	65/69 (94%)	24/71 (34%)	55.13 (10.35–97.83)	>0.001
Tobramycin	57/69 (83%)	16/71 (23%)	50.61 (7.08–37.64)	>0.001
Amikacin	45/70 (64%)	0/71 (0%)	67.04 (−)	>0.001
Gentamycin	56/70 (80%)	13/71 (18%)	53.68 (7.71–41.32)	>0.001
Ciprofloxacin	53/70 (76%)	14/71 (20%)	44.32 (5.70–28.25)	>0.001
Levofloxacin	50/70 (72%)	10/71 (14%)	47.42 (6.54–35.54)	>0.001
Trimethoprim-sulfamethoxazole	17/71 (24%)	25/71 (35%)	2.16 (0.28–1.20)	0.14
Nitrofurantoin	68/69 (98%)	65/71 (92%)	3.61 (0.74–53.57)	0.06

NOTE. Categorical variables are no/total no. (%), CR-KP is carbapenem-resistant *K*. *pneumoniae*, CS-KP is carbapenem-susceptible *K*. *pneumoniae*,. OR is Odds Ratio, 95%CI is Confidence Interval.

### Detection of Antimicrobial Resistance Genes

Among the 71 strains, 62 (87.3%) produced the SHV, 52 (73.2%) produced KPC-2, 18 (25.4%) produced NDM-1, 14 (19.7%) produced CTX-M-15, and 2 (3%) produced IMP-1. None of the isolates produced OXA-48. All ST2390 isolates were both positive for NDM-1 and KPC-2. All ST11 isolates were KPC-2-positive except for one. Among all 71 strains, 19 (26.8%) CR-KP strains harbored ≥3 different resistant genes.

### MLST analysis

ST11 (47) was the most common ST type in this study, followed by ST2390 (5), ST2305 (3), ST736 (2), and the following ST type each represented by one isolate: ST20, ST23, ST25, ST29, ST34, ST147, ST189, ST441, ST629, ST1224, ST1425, ST2236, ST2389, and ST2391. ST2389, ST2390, and ST2391 were identified as novel ST types in the MLST database. More information could be seen on the MLST website (http://bigsdb.pasteur.fr/klebsiella/).

### Strain typing by MALDI-TOF MS

**Figure 2:**
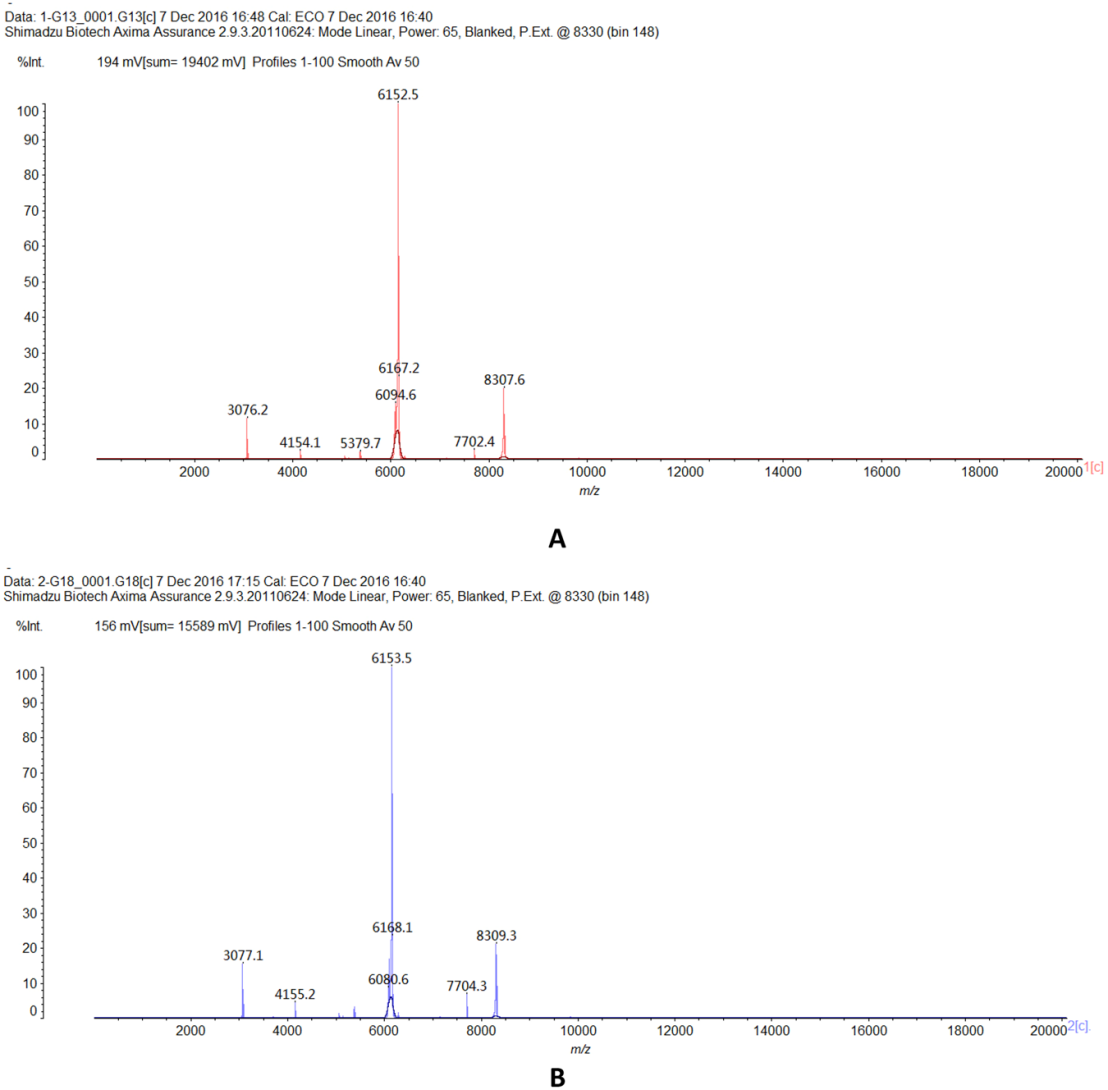
Representative spectra of the carbapenem-resistant *K*. *pneumoniae* strains. A and B show spectra of two representative strains (the strains marked 1 and 2) in our study, respectively.

Even though the 71 clinical strains cultured on the blood agar plate showed diverse morphological characteristics, all of them were correctly identified as *K*. *pneumoniae* species by Clin-TOF II with score values (>25). The representative spectra of the CR-KP strains were shown in Fig. 2.

**Figure 3:**
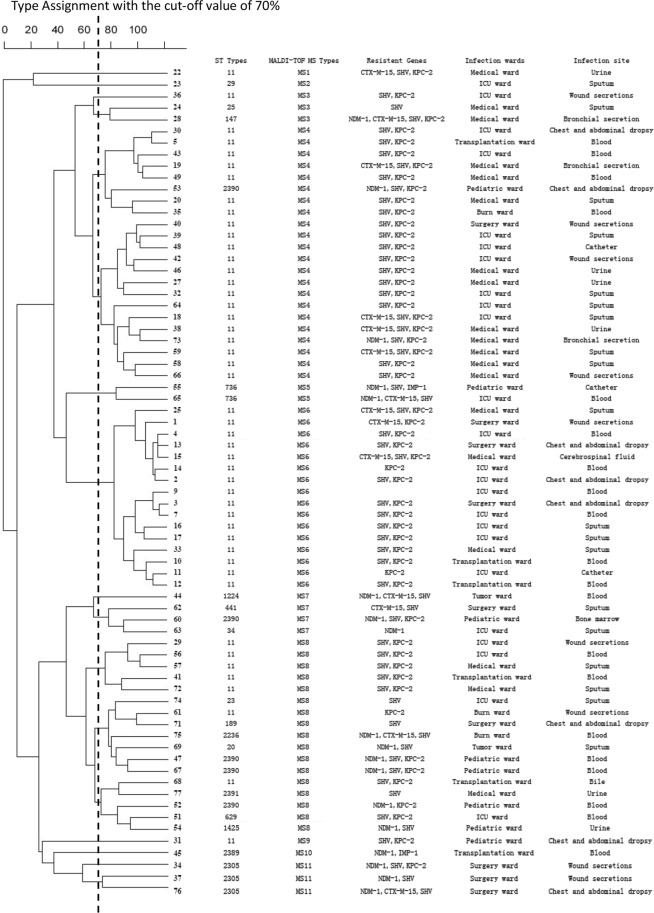
Magnified dendrogram (representation of hierarchical cluster analysis) of the carbapenem-resistant *K*. *pneumoniae*. ST types, matrix-assisted laser desorption/ionization time-of-flight mass spectrometry types, resistant genes, and infection wards of the carbapenem-resistant *K*. *pneumoniae* strains are described.

All 71 clinical strains of *K*. *pneumoniae* were classified into 11 distinct MALDI-TOF MS types: MS1 (1), MS2 (1), MS3 (3), MS4 (22), MS5 (2), MS6 (16), MS7(4), MS8 (17), MS9 (1), MS10 (1), and MS11 (3). Based on the data processed by Clin-TOF II, a dendrogram of MALDI-TOF MS showed clustering of all the clinical strains in diverse partitions. In particular, MS4 and MS6 covered 53.5% of all the MADI-TOF MS types. The rest of the isolates belonged to the ST11 type except strain 53. The dendrogram of the MALDI-TOF MS types, along with the ST types, resistant genes, and the location where the CR-KP strains were isolated were summarized in Fig. 3.

### Temporal Distribution of isolates

**Figure 4:**
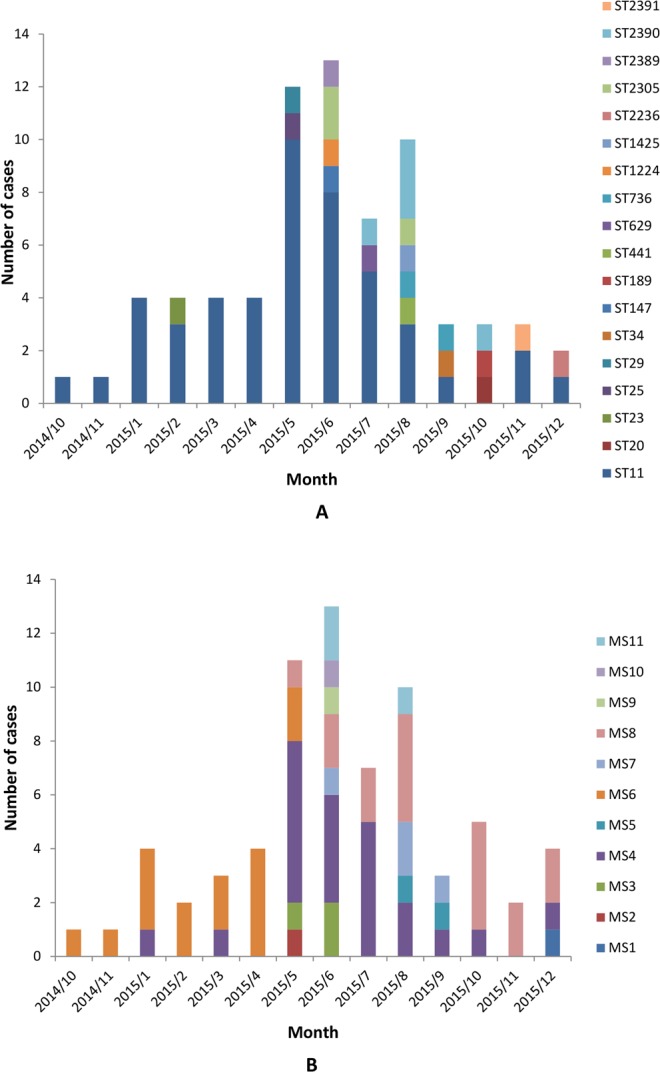
Monthly distribution of multilocus sequence typing (ST) and mass spectrometry types (MS). A shows the distribution of ST types of multilocus sequence typing, ST11cluster and a peak period for outbreak in May and June 2015. B shows the distribution of mass spectrometry (MS) types, mainly MS6, MS4, and MS8 clusters.

A total of 47 ST11 isolates attributed to an outbreak in this study. Most of these strains were isolated from ICU (40.4%) and medical wards (34.0%). During this outbreak, a peak caused by 24 ST11strains (24/47, 51.0%) occurred between May 2015 to August 2015. Over half of the 24 ST11 strains were isolated from patients in ICU (10/24, 41.7%) and medical wards (10/24, 41.7%). Furthermore, MALDI-TOF MS typing also showed an outbreak of MS4 (15/22, 68.2%) within the same timeframe from May 2015 to August 2015, and an outbreak of MS6 (12/15, 80.0%) between November 2014 to April 2015 (Fig. 4).

### Medical Costs of CR-KP infection

The CR-KP infected patients stayed longer in the hospital than the patients with CS-KP infection, meanwhile, the mortality of CR-KP infection patients was higher than that of CS-KP infection patients. Furthermore, the medical costs of CR-KP group (including total costs, medical test costs and total drug costs and anti-infective drug costs) was significantly higher than costs of the CS-KP group (Table 2).

**Table 2:** The mortality and medical costs of carbapenem-resistant KP(CR-KP) and carbapenem-susceptible KP (CS-KP) groups.

	CR-KP (n = 71)	CS-KP (n = 71)	*Z/χ* ^2^	*p*
Mortality (%)	28/71 (39.4%)	16/71 (25.7%)	4.74	0.03
Total costs (¥)	162618 (9098–1078466)	104225 (18145–529492)	−2.87	>0.001
Medical examination	6822 (174–29670)	5077 (284–28496)	−1.09	0.27
Medical test costs (¥)	14124 (1894–74174)	8434 (846–46706)	−2.93	>0.001
Total drug costs (¥)	78579 (965–442989)	38651.5 (8300–222058)	−2.96	>0.001
Anti-infective drug costs (¥)	19755 (63–243121)	7171 (0–63506)	−3.64	>0.001
Total hospital stay days	37 (4–227)	28 (7–149)	−2.33	0.02

NOTE. Continuous variables are median(min-max), CR-KP is carbapenem-resistant *K*. *pneumoniae*, CS-KP is carbapenem-susceptible *K*. *pneumoniae*.

## Discussion

Carbapenem resistance in *Enterobacteriaceae*, especially *K*. *pneumoniae*, has become a significant public health challenge in china. Due to the limited efficacy of antimicrobials in treating carbapenem-resistant *Enterobacteriaceae* (CRE) infection, the mortality of patients infected with CRE is higher than that of patients infected with carbapenem-susceptible *Enterobacteriaceae*(CSE)^16–18^. In a case-control study at a New York City hospital, patients infected with CR-KP showed 48% in-hospital mortality and 38% infection-specific mortality^19^. In this study, patients with CR-KP infection suffered from significant higher mortality, longer hospital stay and higher financial burden compared to patients with CS-KP infection as previously reported^20^.

CR-KP isolates were most frequently isolated from patients from ICU in this study. The first *in vivo* isolation of CR-KP strain was reported in 2000 in an ICU in North Carolina^21^. In fact, ICU was the breeding ground that produced, spread, and amplified antimicrobial resistance because of the presence of extremely vulnerable patients, the use of invasive procedures and the frequent use of antimicrobial agents^22,23^. Published literature reported that upon admission to the ICU, 13% of the patients were already colonized with KPC-KP^24^, and up to 74.5% of the patients were reported to be colonized with KPC-KP during their stay at the ICU^25^.

In China, *Klebsiella pneumoniae* carbapenemase (KPC) is the most clinically significant serine carbapenemase. KPC-2-producing *K*. *pneumoniae* isolates spread widely and rapidly across the country, after the first KPC-2-producing *K*. *pneumoniae*(KPC-KP) was isolated in China^26^. In our study, most of the CR-KP strains were KPC-2-producing *K*. *pneumoniae*, which were major hospital pathogens^27,28^. Further, 18 strains harbored the NDM-1 carbapenemase gene, which made the strain confer resistance to almost all β-lactams, except aztreonam^29^. NDM-producing isolates were usually resistant to multiple antimicrobials, leaving few or no therapeutic options^30^. Meanwhile, 14 strains harbored CTX-M-15 Extended-Spectrum β-Lactamases (ESBL) genes, which was globally the most prevalent variant in the CTX-M variants^31^. CTX-M enzymes have emerged as a predominant type of ESBL produced by clinical isolates of *Enterobacteriaceae* in the world^32^.

Our study presented that 26.8% (19/21) CR-KP harbored ≥3 different resistance- associated genes. This was consistent with the finding of Li B, *et al*.^33^. Of note, multi-carbapenemase production is associated with multi-drug resistance, which leads to limited anti-infection treatment options. We have surveillance systems for multiple drug resistant (MDR) bacteria in the hospital. Patients with MDR bacteria would be isolated in hospital, and workers who had contacted the patient would enhance hand hygiene. Once an outbreak was detected, we should notify the infection departments to isolate hospitalization with MDR infection, and strengthen disinfection.

Our study provided information on clinical characteristics and molecular epidemiology of CR-KP infection in central China by typing isolates from an outbreak using MLST and MALDI-TOF MS. In this study, ST11was the predominant strain attributed to the outbreak. ST11 is the epidemic ST type of KPC-producing *K*.*pneumoniae* in China^34^, and almost all ST11 isolates were KPC-2-producing *K*.*pneumoniae*, contributing to the spread of antibiotic resistance in the hospitals. In addition, almost all ST11 isolates were matched with MS4 and MS6 in MS typing, which covered 53.5% of all the MADI-TOF MS types. During the outbreak described in this study, over half of the ST11 isolates were distributed from May 2015 to August 2015, most of which were MS4 in MALDI-TOF MS typing. Hierarchical cluster analysis of strains by MALDI-TOF MS was acquired and analyzed simultaneously with the antimicrobial sensitivity results. The quick identification of an outbreak was critical for infection control.

In summary, an outbreak of KPC-2-producing CR-KP isolates was reported in our hospital, which was associated with higher financial burden and longer hospital stay. ST11 isolates were the predominant ST type attributed to the outbreak, and most of these isolates were matched with MS4 and MS6 in MS typing. MALDI-TOF MS typing can rapidly identify and type the CR-KP isolates. This is the first report of the utilization of MALDI-TOF MS in understanding the lineage of isolates contributing to CR-KP outbreak in china. This is also the first study that evaluates the financial burden of CR-KP infection in china. Findings of this study will help establish Antimicrobial Stewardship Program (ASP) and develop HAI outbreak surveillance, prevention and control programs on CR-KP infection in China.

This study presents several limitations. First, because of the restrictions on technology and funds, PFGE and *wzi* gene sequencing was not conducted. Moreover, this study was conducted in a single medical center, the sample size of the CR-KP group and CS-KP group was small for the risk factor assessment. Study on a larger population may produce more comprehensive results, and patient-to-patient transmission of HAI caused by CRKP was not assessed. Instead, MALDI-TOF MS typing was utilized for its short turnaround time which may have produced a relatively rough description of outbreak^35^.

## Material and Methods

### Study Setting and Bacterial Isolates

This was a retrospective study carried out in Xiangya Hospital, a 3,500-bed university teaching hospital in Changsha, Hunan Province, Central South China. This hospital provides medical and surgical care for all patients including adults and children.

All non-duplicate bacterial isolates were collected from clinical samples from October 2014 to December 2015. Isolates recovered from the same patients were counted only once. Patients’ medical records were retrospectively reviewed and all data collected were de-identified.

### Strain Identification and Antimicrobial Susceptibility Testing

The Vitek 2 system (bioMérieux, Marcy l'Étoile, France) was used for the identification of bacterial isolates. Antibiotic susceptibility was tested by microbroth dilution to determine the minimum inhibitory concentration (MIC) of the antimicrobials (including imipenem, meropenem, ertapenem, cefoxitin, amoxillin/clavulanic acid, piperacillin/tazobactam, ampicillin/sulbactam, cefazolin, ceftazidime, ceftriaxone, nitrofurantoin,cefepime, aztreonam, cefotetan, cefoperazone/sulbactam, tobramycin, gentamycin, ciprofloxacin, levofloxacin, amikacin, trimethoprim-sulfamethoxazole). The MICs of different antimicrobials were interpreted using the EUCAST breakpoints standards from 2014 (http://www.eucast.org/clinical_breakpoints/). *Escherichia coli* ATCC25922 and *Pseudomonas aeruginosa* ATCC27853 were used as controls.

### Detection of Antibiotic Resistance Genes

Antibiotic resistance genes were detected by polymerase chain reaction (PCR) using primers and conditions as previously described^33,36^. PCR was performed for all the CR-KP strains to detect the carbapenemase genes (*bla*_NDM-1_, *bla*_KPC-2_, *bla*_IMP-1_, and *bla*_OXA-48_) and β-lactamases genes (*bla*_CTX-M-15_ and *bla*_SHV_). PCR products were purified and sequenced using the amplification primers at Sangon Biotech (Shanghai, China).

### MLST analysis

PCR amplification of seven housekeeping genes (*gapA*, *infB*, *mdh*, *pgi*, *phoE*, *rpoB*, and *tonB*)were performed on all CR-KP isolates as previously described^37^. The allele number and sequence type (ST) were assigned by MLST website (http://bigsdb.pasteur.fr/klebsiella/).

### Strain Typing by MALDI-TOF MS

For each isolate, three separate spectra were obtained using the measurements performed on Clin-ToF II (Bioyong Technologies Inc, Beijing). *Escherichia coli* ATCC8739 was used for the calibration of the instrument. All spectra were recorded in the linear positive-ion mode at a laser frequency of 40 Hz and mass ranging from 2,000 to 20,000 kDa. For each sample spot, one spectrum was acquired as a sum of 500 shots across a spot. Species were confirmed by comparison with the mass-spectrum library, using the BioExplorer V2.1 DataBase (Bioyong Technologies Inc) under standard conditions. The basal MALDI-TOF MS classification data were obtained from three points with the highest degree. The data with the highest degree were selected for cluster analysis.

MALDI-TOF results were analyzed using MALDI MS software (Bioyong Technologies Inc, Beijing). The differences of *K*. *pneumoniae* MS were analyzed using the Launch pad software (Bioyong Technologies Inc, Beijing). All peak spectra and positions were compared with 1/1000 m/z offset value. Matrix was constructed with m/z peak position, and hierarchical clustering was analyzed through Flashclust software (Bioyong Technologies Inc, Beijing). The differences in homology were set using the perimeter distance of a dendrogram. According to the type assignment, we defined a cut-off value was >70% similarity.

### Clinical Data Collection and Definitions

CR-KP strains were defined as isolates with resistance or intermediate sensitivity to at least one type of carbapenem (imipenem, ertapenem, or meropene). For each CR-KP infected patient, one patient with CS-KP infection was randomly selected. The two groups were admitted within the same period (within 30 days) and matched for age and sex. The clinical data of these patients were retrospectively reviewed.

Total cost was defined as all costs of the patients in hospital; medical examination cost was defined as the cost associated with examining the patients, including imaging and other laboratory tests; medical testing cost was defined as the cost of laboratory testing; drug cost was defined as the cost of all medications; and anti-infective drug cost was defined as the cost of antimicrobial agents.

### Statistical Analysis

All the statistical analyses were performed using SPSS 20.0 (IBM). The categorical variables were expressed by rates and tested by Chi-square. The Wilcoxon Rank SumTest was used to compare the continuous variables which were shown as median. Two-tailed *P* value of less than 0.05 was considered significant.

### Ethics statement

All procedures performed in this study involving human participants were in accordance with the Ethics Committee of the Xiangya Hospital of Central South University (No. 201701017). The study was conducted in accordance with the Declaration of Helsinki. Oral informed consent was obtained from all individual participants included in the study. This article does not contain any studies with animals performed by any of the authors.

## Data Availability

The datasets supporting the conclusions of this article are included within the article. The raw data can be made available to the interested researchers by the authors of this article if requested.

## References

[1] Ahmad TA, Haroun M, Hussein AA, El Ashry el SH, El-Sayed LH (2012). Development of a new trend conjugate vaccine for the prevention of Klebsiella pneumoniae. Infectious disease reports..

[2] Neuhauser MM (2003). Antibiotic resistance among gram-negative bacilli in US intensive care units: implications for fluoroquinolone use. Jama..

[3] Orsi GB (2011). Risk factors and clinical significance of ertapenem-resistant Klebsiella pneumoniae in hospitalised patients. The Journal of hospital infection..

[4] Won SY (2011). Emergence and rapid regional spread of Klebsiella pneumoniae carbapenemase-producing Enterobacteriaceae. Clinical infectious diseases..

[5] Tzouvelekis LS, Markogiannakis A, Psichogiou M, Tassios PT, Daikos GL (2012). Carbapenemases in Klebsiella pneumoniae and other Enterobacteriaceae: an evolving crisis of global dimensions. Clinical microbiology reviews..

[6] Munoz-Price LS (2013). Clinical epidemiology of the global expansion of Klebsiella pneumoniae carbapenemases. The Lancet. Infectious diseases..

[7] Li P (2017). ST37 Klebsiella pneumoniae: development of carbapenem resistance *in vivo* during antimicrobial therapy in neonates. Future Microbiology.

[8] Jian Z (2014). Detection of the novel IMP-38 among carbapenemase-producing Enterobacteriaceae in a university hospital, China[J]. Journal of Infection in Developing Countries.

[9] Hu L (2014). The prevalence of carbapenemase genes and plasmid-mediated quinolone resistance determinants in carbapenem-resistant Enterobacteriaceae from five teaching hospitals in central China. Epidemiology and infection..

[10] Qin X, Yang Y, Hu F, Zhu D (2014). Hospital clonal dissemination of Enterobacter aerogenes producing carbapenemase KPC-2 in a Chinese teaching hospital. Journal of medical microbiology..

[11] Zhang Y (2014). Contribution of beta-lactamases and porin proteins OmpK35 and OmpK36 to carbapenem resistance in clinical isolates of KPC-2-producing Klebsiella pneumoniae. Antimicrobial agents and chemotherapy..

[12] Xiao YH (2011). Epidemiology and characteristics of antimicrobial resistance in China. Drug resistance updates..

[13] Zheng B (2017). Molecular Epidemiology and Risk Factors of Carbapenem-Resistant Klebsiella pneumoniae Infections in Eastern China. Frontiers in microbiology..

[14] Amiri-Eliasi B, Fenselau C (2001). Characterization of protein biomarkers desorbed by MALDI from whole fungal cells. Analytical chemistry..

[15] Hu YY, Cai JC, Zhou HW, Zhang R, Chen GX (2015). Rapid detection of porins by matrix-assisted laser desorption/ionization-time of flight mass spectrometry. Frontiers in microbiology..

[16] Hussein K (2013). Impact of carbapenem resistance on the outcome of patients’ hospital-acquired bacteraemia caused by Klebsiella pneumoniae. The Journal of hospital infection..

[17] Li C (2014). Point-prevalence of healthcare-associated infection in china in 2010: a large multicenter epidemiological survey. Infection control and hospital epidemiology..

[18] Meng X (2017). Risk factors and medical costs for healthcare-associated carbapenem-resistant Escherichia coli infection among hospitalized patients in a Chinese teaching hospital. BMC infectious diseases..

[19] Patel G, Huprikar S, Factor SH, Jenkins SG, Calfee DP (2008). Outcomes of carbapenem-resistant Klebsiella pneumoniae infection and the impact of antimicrobial and adjunctive therapies. Infection control and hospital epidemiology..

[20] van Duin D (2014). Surveillance of carbapenem-resistant Klebsiella pneumoniae: tracking molecular epidemiology and outcomes through a regional network. Antimicrobial agents and chemotherapy..

[21] Yigit H (2001). Novel carbapenem-hydrolyzing beta-lactamase, KPC-1, from a carbapenem-resistant strain of Klebsiella pneumoniae. Antimicrobial agents and chemotherapy..

[22] Brusselaers N, Vogelaers D, Blot S (2011). The rising problem of antimicrobial resistance in the intensive care unit. Annals of intensive care..

[23] Li C (2013). Changes in antimicrobial use prevalence in China: results from five point prevalence studies. PloS one..

[24] Papadimitriou-Olivgeris M (2012). Risk factors for KPC-producing Klebsiella pneumoniae enteric colonization upon ICU admission. The Journal of antimicrobial chemotherapy..

[25] Papadimitriou-Olivgeris M (2013). KPC-producing Klebsiella pneumoniae enteric colonization acquired during intensive care unit stay: the significance of risk factors for its development and its impact on mortality. Diagnostic microbiology and infectious disease..

[26] Wei ZQ (2007). Plasmid-mediated KPC-2 in a Klebsiella pneumoniae isolate from China. Antimicrobial agents and chemotherapy..

[27] Jain R (2013). Emergence of Carbapenemaseproducing Klebsiella Pneumoniae of Sequence type 258 in Michigan, USA. Infectious disease reports..

[28] Chen YT (2014). KPC-2-encoding plasmids from Escherichia coli and Klebsiella pneumoniae in Taiwan. The Journal of antimicrobial chemotherapy..

[29] Principe L (2017). First report of NDM-1-producing Klebsiella pneumoniae imported from Africa to Italy: Evidence of the need for continuous surveillance. Journal of global antimicrobial resistance..

[30] Nordmann P, Naas T, Poirel L (2011). Global spread of Carbapenemase-producing Enterobacteriaceae. Emerging infectious diseases..

[31] Pitout J. D. & Laupland K. B., Extended-spectrum beta-lactamase-producing Enterobacteriaceae: an emerging public-health concern. *The Lancet*. *Infectious diseases*. **8**, 159–166 (Mar, 2008). 10.1016/S1473-3099(08)70041-0 18291338

[32] Canton R, Coque TM (2006). The CTX-M beta-lactamase pandemic. Current opinion in microbiology..

[33] Li B (2012). Analysis of drug resistance determinants in Klebsiella pneumoniae isolates from a tertiary-care hospital in Beijing, China. PloS one..

[34] Li JJ (2012). Epidemic of Klebsiella pneumoniae ST11 clone coproducing KPC-2 and 16S rRNA methylase RmtB in a Chinese University Hospital. BMC infectious diseases..

[35] Brisse S (2013). wzi Gene sequencing, a rapid method for determination of capsular type for Klebsiella strains. Journal of clinical microbiology..

[36] Zhuo C, Li XQ, Zong ZY, Zhong NS (2013). Epidemic plasmid carrying bla(CTX-M-15) in Klebsiella penumoniae in China. PloS one..

[37] Diancourt L, Passet V, Verhoef J, Grimont PA, Brisse S (2005). Multilocus sequence typing of Klebsiella pneumoniae nosocomial isolates. Journal of clinical microbiology..

